# Household Transmission of Gastroenteritis

**DOI:** 10.3201/eid1107.040889

**Published:** 2005-07

**Authors:** Sharon Perry, Maria de la Luz Sanchez, Philip K. Hurst, Julie Parsonnet

**Affiliations:** * Stanford University School of Medicine, Stanford, California, USA

**Keywords:** acute gastroenteritis, infectious diarrhea, household transmission

## Abstract

Transmission of infectious gastroenteritis was studied in 936 predominately Hispanic households in northern California. Among 3,916 contacts of 1,099 primary case-patients, the secondary attack rate was 8.8% (95% confidence interval 7.9–9.7); children had a 2- to 8-fold greater risk than adults. Bed-sharing among children in crowded homes is a potentially modifiable risk.

Infectious diarrhea poses a major problem for the US healthcare system and employers. Although <5% of episodes may result in a physician encounter (1), surveillance systems have estimated that 0.72 episodes occur per person-year, and up to 1.1 episodes per year for children <5 years of age (2). Household transmission of infectious gastroenteritis is likely to account for a substantial portion of community incidence. With the exception of a few prospective studies (3,4), studies of household transmission of gastroenteritis have typically reported on community outbreaks of individual pathogens followed up in the home (5–9). In these outbreak settings, secondary attack rates were 4%–20%, depending on pathogen, mode of transmission, and length of time spent in the household. Since household clusters of gastroenteritis may parallel larger community trends (10), information about baseline incidence and risk factors is useful to validate population-based and sentinel surveillance systems.

## The Study

Index cases of probable infectious gastroenteritis were identified through 15 participating community health providers; 11 of these were public health clinics serving low-income families. After an initial telephone interview, a home visit was scheduled within 2 weeks of the index episode. Household contacts of the index patient were interviewed regarding symptoms and onset of diarrheal illness. A second visit 3 months later completed documentation of the household episode. The cohort consists predominately of Hispanic families with young children, including a median of 5 persons (range 2–20) per household and a median sleeping density of 2.5 persons/bedroom. Households were excluded if they had <2 participating members or if the living unit was a dormitory or other communal residential arrangement.

Infectious gastroenteritis was defined as 1) diarrheal illness lasting <14 days and marked by symptoms of loose or watery stool occurring at least 5 times per day in a child <2 years of age, or at least 3 times per day in older persons or 2) at least 1 instance of vomiting per day in a person of any age. Illnesses considered noninfectious in origin, such as those due to morning sickness, poisoning, medications, or alcohol, were excluded from the case definition. A primary case was defined as the first household case with onset within 2 weeks of the index referral or onset within 2 days of the first primary case. Secondary gastroenteritis was defined as an illness meeting the case definition of infectious gastroenteritis, beginning at least 2 days after the onset and ≤5 days after the end of an episode in another household contact. A household episode was deemed to have concluded when all members had been symptom-free for at least 5 days. Secondary attack rates were estimated crudely and also modeled by using the life-table method. Risk factors associated with secondary transmission were assessed with logistic regression, with an exchangeable correlation matrix to account for household clusters.

From 1999 to 2004, a total of 3,747 index referrals were received; 2,094 (56%) persons could be contacted by phone and met initial eligibility criteria. Of these, 830 (40%) declined participation, and 1,264 (60%) were scheduled for a home visit. After the initial home visit, 1,154 households were enrolled, and 102 were excluded because the index episode did not meet the case definition (n = 80), the household had <2 interested members (n = 20), or someone in the household had participated in a prior study (n = 2). Of the 1,154 households enrolled, 936 (81%) completed documentation of ≥1 household gastroenteritis episode. These 936 households had 5,783 members. Of these, 5,015 (87%) gave reports sufficient to classify symptoms as primary, secondary, or absent; 557 (9.6%) did not participate in interviews; and 211 (3.6%) reported diarrhea, vomiting, or both that could not be classified temporally. Of participating members, 24% were ≤5 years of age, 18% were 6–17 years of age, and 58% were ≥18 years of age.

**Figure 1:**
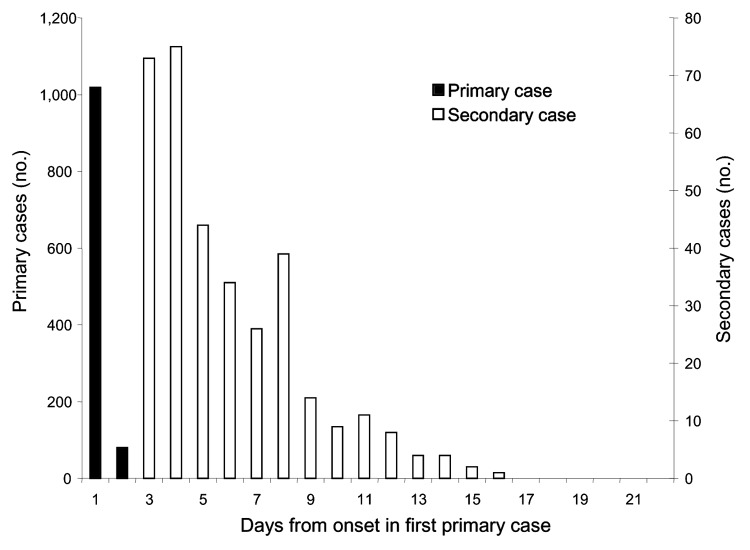
Serial onset of 344 secondary cases in 936 households. A secondary case was defined as onset of symptoms at least 2 days after onset and not more than 5 days after cessation of symptoms in a primary case.

Household episodes lasted a median of 9 days (range 5–29 days) and involved 1–6 household members. Of the 1,443 (29%) household members who reported symptoms consistent with infectious gastroenteritis, 1,099 (76%) were classified as primary and 344 (24%) as secondary case-patients (Figure 1). Median age of primary cases was 3.6 years, including 60% <5 years of age (Table 1).

**Table 1:** Characteristics of 1,099 primary case-patients and 3,916 household contacts

	Primary (n = 1,099) (%)	Secondary (n = 344) (%)	No symptoms (n = 3,572) (%)
No. persons in household, median	5*	6	5*
<18 y	2	3	2
≥18 y	3	3	3
Index case	852 (78)*	79 (23)	–
Member/index family	1,052 (96)*	295 (86)	2,064 (58)*
Age, y, median	3.6*	12.6	24.7*
<2	412 (37)	57 (17)	138 (4)
2–5	254 (23)	50 (15)	303 (8)
6–17	145 (13)	76 (22)	670 (19)
≥18	288 (26)	161 (47)	2,461 (69)
Male	552 (50)*	148 (43)	1,803 (50)*
Daycare (if <6 y)	148 (22)	24 (22)	108 (24)
Symptoms			
Duration, median days	3*	1.3	–
Vomiting with or without diarrhea	799 (73)*	194 (56)	–
Shares a bed with primary case-patient	–	152 (44)	776 (22)*
Exposed to vomiting, primary case-patient	–	87 (25)	649 (18)*

*Univariate analysis, p<0.05 for comparison of secondary and primary cases, or secondary cases and other contacts of primary cases; –, not applicable.

**Figure 2:**
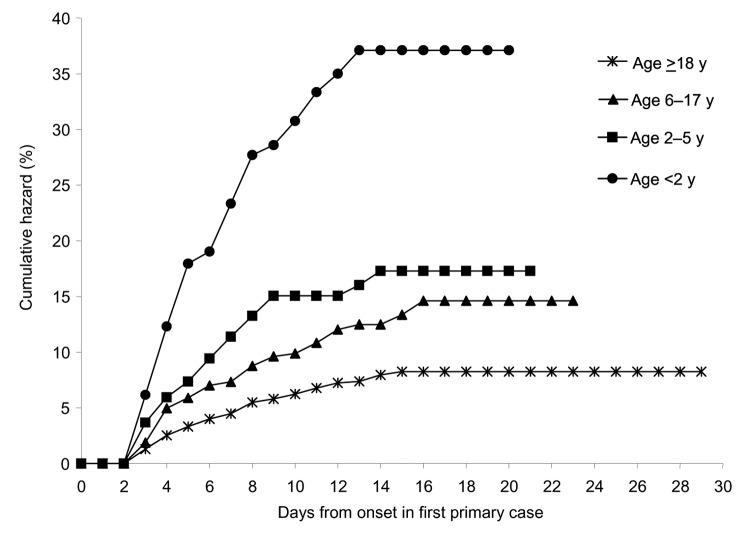
Hazard of secondary gastroenteritis by age group. Cumulative hazard, the cumulative proportion of contacts classified as secondary cases. Household risk periods, defined as ending when all members had been symptom-free for ≥96 hours, lasted a median of 9 days (interquartile range 7–13).

Among 3,916 contacts of the 1,099 primary case-patients, the crude secondary attack rate was 8.8% (95% confidence interval [CI] 7.9–9.7). Household transmission occurred within a median of 4 days (range 2–15 days) of onset of symptoms in the first primary case. Cumulative hazard rates varied substantially by age: 37%, 17%, 15%, and 8% for study participants <2, 2–5, 6–17, and ≥18 years of age, respectively (Figure 2). Secondary transmission occurred in 240 (26%) homes, and households with secondary cases were larger, with a median of 3 versus 2 children.

The crude secondary attack rate of 9% is somewhat lower than, but not inconsistent with, prior estimates. Pickering et al. (5) reported an overall secondary attack rate of 11% among family members of children involved in daycare outbreaks, with rates of 26%, 15%, and 17% for *Shigella*, rotavirus, and *Giardia*, respectively. After a foodborne outbreak of Norovirus, Gotz et al. (6) estimated that 20% of family members of daycare participants became ill. In an investigation of sporadic *Escherichia coli* O157, Parry et al. (9) estimated attack rates of 4%–15% in household contacts. In a small Danish community, 12% of family members became ill after children attending a daycare center in a neighboring town were exposed to a contaminated water supply (11). Similarly, 11% of family members became ill within 3 days of children's return from summer camp, where they were exposed to a suspected viral agent (8).

Since outbreak and surveillance investigations typically focus on highly transmissible agents with more severe illnesses, the somewhat lower secondary attack rate observed in this study of unidentified, mixed agents is not surprising. Risk interval (ours including a 96-hour postsymptomatic period) may affect classification of secondary illness. Among 835 households completing the follow-up visit, 380 (46%) had up to 7 recurrent episodes during a 3-month period, 42 beginning 6–10 days after conclusion of the first episode. Merging these episodes would have increased crude attack rates slightly, to ≈10%. Conversely, >80% of secondary cases occurred within 7 days of primary onset. Thus, our risk interval was likely to capture incubation periods of more common causal agents in the United States, although the fact that these homes were susceptible to recurrent episodes over time cannot be ignored.

The risk factor analysis (Table 2) confirmed the role of age in household transmission; children exhibited a 2- to 8-fold greater risk for secondary gastroenteritis compared with adults. Although 60% of patients with primary cases were <5 years of age, secondary transmission was more likely when the primary case-patient was >5 years (adjusted odds ratio [AOR] 1.7 [95% CI 1.3–2.3]). In addition, being a member of the index family (AOR 2.5 [95% CI 1.7–3.6]) and sharing a bed with a primary case-patient (AOR 2.0 [95% CI 1.5–2.6]) were independently associated with risk. As observed in some studies of Norovirus (6,12), exposure to vomiting was also associated with household transmission (AOR 1.6 [95% CI 1.2–2.2]), despite the fact that only 1 of 5 contacts reported this exposure.

**Table 2:** Factors associated with secondary gastroenteritis among 3,916 household contacts

Characteristic	Adjusted odds ratio (95% CI)*	p value*
Age, y		
<2	8.0 (5.5–11.4)	<0.0001
2–5	3.0 (2.0–4.3)	<0.0001
6–17	2.0 (1.5–2.8)	<0.0001
≥18	Reference	
Shares bed with primary case-patient	2.0 (1.5-2.7)	<0.0001
Exposure/primary vomiting episode	1.6 (1.2–2.2)	0.001
Member of index family	2.5 (1.7–3.6)	<0.0001
Primary cases, no.	1.5 (1.0–2.2)	0.07
Primary cases, ≥5 y	1.7 (1.3–2.3)	0.0009
Primary cases, duration, days	1.09 (1.0–1.1)	<0.0001

*p values and confidence intervals (CI) obtained by logistic regression for correlated observations, assuming exchangeable correlation within households.

## Conclusions

Viral agents are thought to account for 80% of reported community diarrhea (13), and repeated exposure to agents like rotavirus, as is likely to occur in homes with small children, may be associated with features of acquired immunity (14). Some support for this hypothesis is the fact that, compared with primary cases, secondary cases tended to be in older children, with shorter episodes and fewer vomiting episodes (Table 1). Conversely, children <5 years of age, who constituted nearly one fourth of household members and had more than half of all illnesses, had more protracted episodes, regardless of primary or secondary onset or symptoms. Thus, although age-related immunity may have played a role in modifying duration or severity of secondary illness, the high proportion of young children was more determinative of household risk. Bed-sharing with these children was likely the major factor in propagating infection in these crowded homes.

Although our enrollment rate is similar to that in other population-based studies of gastroenteritis (1,15), we cannot exclude the possibility that participating families had different attack rates than nonparticipants. Approximately 13% of members in participating households were not interviewed or could not be classified as to temporal onset of symptoms; however, ascertainment did not differ between households with and without secondary cases. Although 19% of households were lost to follow-up, most commonly because of relocation, attack rates among completing households available for 1 visit or 2 were not significantly different. Our risk factor model, which assumes that multiple events within households share a common correlation across time, may be strong for longitudinal data, although for more limited risk periods and conditions of intense, close contact, the approach may be plausible.

In conclusion, household transmission continues to play an important role in community rates of acute intestinal infections. Bed-sharing among children in crowded homes constitutes a potentially modifiable risk.
